# ALCAM regulates multiple myeloma chemoresistant side population

**DOI:** 10.1038/s41419-022-04556-8

**Published:** 2022-02-10

**Authors:** Fangfang Wang, Zhang Dan, Hongmei Luo, Jingcao Huang, Yushan Cui, Hong Ding, Juan Xu, Zhimei Lin, Yuhan Gao, Xinyu Zhai, Yan Yang, Ying Qu, Li Zhang, Fengjiao Chen, Qiang Wang, Xin Wang, Yu Feng, Ting Liu, Qing Yi, Ting Niu, Yuhuan Zheng

**Affiliations:** 1 Department of Hematology, West China Hospital/State Key Laboratory of Biotherapy and Cancer Center, Sichuan University, Chengdu, China; 2 Department of Hematology, The Affiliated Hospital of Chengdu University, Chengdu, China; 3 Center for Translational Research in Hematological Malignancies, Cancer Center, Houston Methodist Hospital, Houston, TX USA

**Keywords:** Cancer microenvironment, RNAi

## Abstract

Drug-resistance is a major problem preventing a cure in patients with multiple myeloma (MM). Previously, we demonstrated that activated-leukocyte-cell-adhesion-molecule (ALCAM) is a prognostic factor in MM and inhibits EGF/EGFR-initiated MM clonogenicity. In this study, we further showed that the ALCAM-EGF/EGFR axis regulated the MM side population (SP)-mediated drug-resistance. ALCAM-knockdown MM cells displayed an enhanced ratio of SP cells in the presence of bone marrow stromal cells (BMSCs) or with the supplement of recombinant EGF. SP MM cells were resistant to chemotherapeutics melphalan or bortezomib. Drug treatment stimulated SP-genesis. Mechanistically, EGFR, primed with EGF, activated the hedgehog pathway and promoted the SP ratio; meanwhile, ALCAM inhibited EGFR downstream pro-MM cell signaling. Further, SP MM cells exhibited an increased number of mitochondria compared to the main population. Interference of the mitochondria function strongly inhibited SP-genesis. Animal studies showed that combination therapy with both an anti-MM agent and EGFR inhibitor gefitinib achieved prolonged MM-bearing mice survival. Hence, our work identifies ALCAM as a novel negative regulator of MM drug-resistance, and EGFR inhibitors may be used to improve MM therapeutic efficacy.

## Introduction

Multiple myeloma (MM) is the second most common hematologic malignancy in the US [1]. In the new drug era, with the landmark clinical application of proteasome inhibitors (PIs) and immunomodulatory drugs (IMiDs) in MM treatment, MM is treatable but remains an incurable disease [2]. Drug-resistance widely exists, particularly in some newly diagnosed high-risk MM patients or relapsed/refractory MM patients [3]. However, the mechanism of MM drug-resistance is still not fully understood.

More than a decade ago, Matsui et al. reported heterogeneous cell types in an MM cell line with distinguishable clonogenicity activities [4]. A small population of MM cells, usually <2% of the total tumor cells, with clonogenicity capacity was referred to as myeloma cancer stem cells (MM-CSCs) in the same study. Later, the self-renewal, differentiation, and drug-resistant features of MM-CSCs have been demonstrated in many basic studies [5]. MM-CSCs may be identified by different methods, and no consensus of MM-CSC markers or detection methodology currently exists [6]. Hoechst staining is often used to separate MM cells into a side population (SP) and a main population (MP), and the SP MM cells are believed to contribute to MM clonogenesis; therefore, SP MM cells are considered to have “stemness.” [7] The drug-resistance feature associated with SP MM cells has also been investigated [8, 9]. More importantly, a recent study using clinical samples suggested a relationship between the stemness features of SP MM cells and MM minimal residual disease [10]. In this work, we investigated the function of activated-leukocyte-cell-adhesion-molecule (ALCAM, also known as CD166) in SP MM cell regulation.

ALCAM is a member of immunoglobulin superfamily proteins [11]. Recently, we demonstrated the ALCAM function in myelomagenesis [12]. ALCAM negatively regulates myeloma clonogenicity. MM patients with high ALCAM expression have superior overall survival. Mechanistically, ALCAM interacts with EGFR and inhibits EGFR downstream pro-MM cell signaling. In this study, we showed that ALCAM regulated MM drug-resistant SP MM cells. Targeting the ALCAM-EGFR/EGF axis might therefore overcome SP MM-mediated drug-resistance.

## Materials and methods

### Primary myeloma samples

Bone marrow (BM) aspirations from newly diagnosed MM patients were provided by the tissue bank of the Department of Hematology, West China Hospital, Sichuan University, and processed as previously described [12]. This study was approved by the Ethical Committee of West China Hospital of Sichuan University.

### Cell culture

Human MM cell lines RPMI8226 and MM.1S were maintained in RPMI-1640 medium with 10% fetal bovine serum (Gemini BioProducts, US), 100 units/mL penicillin, and 100 g/mL streptomycin at 37 °C and 5% CO_2_. Murine MM cell line 5TGM1 with consistent luciferase gene expression was maintained in the same culture condition. The cell lines were verified by short tandem repeat analysis and tested for mycoplasma contamination. To generate MM cells with a consistently low ALCAM expression, human MM cell lines RPMI8226 and MM.1S were infected with two different ALCAM shRNA lentiviruses (#TLHVU2300, Transomic Tech., US).

The following oligonucleotides were used as shRNA sequences to target ALCAM (sh1 5′-CAGAGGAATCTCCTTATATA-3′ and sh2 5′-CCGAAGGAATAAGAAGCTCAA-3′). Infected cells were selected and maintained in the culture medium with the addition of puromycin (Sigma Aldrich, US). The control viruses were ordered from Transomic Tech (#TLHVU2300, Transomic Tech., US). Human bone marrow stromal cells (BMSCs) were derived and maintained as previously described [12].

### Antibodies and reagent

Anti-ALCAM/CD166 (#343905) antibody for flow cytometry analysis was ordered from Biolegend Inc. Recombinant proteins, including human ALCAM-Fc (#CD6-H5259) and EGF (#10605-HNAE), were ordered from Acrobiosystems and Sinobiology Inc., respectively. Anti-EGF (#AF236) and anti-CD6 (#AF627) neutralizing antibodies were ordered from R&D Systems Inc. EGFR inhibitor gefitinib (#S1025), SMO inhibitor cyclopamine (#S1146), and Gli1 inhibitor GANT61 (#S8075) were ordered from Selleck Inc. Two mitochondrial respiratory chain inhibitors—oligomycin (#C3007) and rotenone (#B5462)—were ordered from ApexBio Technology. Melphalan (#148-82-3) and bortezomib (#179324-69-7) were ordered from MedChemExpress.

### Colony formation assay

The soft agar colony formation assay was performed as previously described [12].

### Flow cytometry analysis

Hoechst staining, followed by flow cytometry analysis, was performed as previously described [13]. In brief, cells were collected and re-suspended at a density of 1 × 10^6^ cells/mL in DMEM supplemented with 2% heat-inactivated fetal bovine serum (FBS) and 10 mM HEPES, pre-warmed to 37 °C. Hoechst 33342 was added at a concentration of 5 μg/mL. In a parallel sample aliquot, SP inhibitor verapamil (#V4629, Sigma Aldrich LLC, US) was used at a final concentration of 5 μM. Cells were incubated for 2 h in a water bath at 37 °C with periodic agitation. Cells were then centrifuged for 5 min at 400×*g*, 4 °C, and resuspended at a concentration of 1 × 10^7^ cells/mL in cold HBSS containing 2% FBS and 10 mM HEPES. Propidium iodide (PI) was added to a final concentration of 5 μg/mL to gate-off dead cells. SP cells were examined by flow cytometry (Moflo XDP, Beckman Coulter, US). In some experiments, SP cells were sorted with the same instrument; the same was also true for MP cells’ sorting.

An ALDEFLUOR assay was performed with an ALDEFLUOR kit (#01700, STEMCELL Tech.) to examine the aldehyde dehydrogenase (ALDH) activity of cells. Cells were collected and re-suspended at a density of 1 × 10^6^ cells/mL in an ALDEFLUOR™ assay buffer, and 5 μL of the activated ALDEFLUOR™ reagent was added per milliliter of the sample. In a parallel sample tube, 5 μL ALDEFLUOR™ DEAB reagent was added as a control. Cells were incubated for 30 min at 37 °C, centrifuged for 5 min at 400×*g*, 4 °C, and re-suspended in 0.5 mL of cold ALDEFLUOR™ assay buffer. Samples were analyzed by Navios flow cytometer (Beckman Coulter, USA).

For cell-cycle analysis, the cells were collected and fixed in ice-cold 70% ethanol at 4 °C overnight. Then, the cells were centrifuged and washed with PBS, and the pelleted cells were incubated with 1 μg/mL propidium iodide (PI) solution containing 100 μg/mL RNase at 37 °C for 30 min. The percentage of cells in each cell-cycle phase was quantified using Modfit software (Verity Software House). For the flow cytometry-based apoptosis examination, the cells were harvested, washed, and re-suspended in 100 µL of binding buffer. Apoptosis cells were examined using an Annexin V-FITC/PI apoptosis kit (#FXP018-100, 4A Biotech, China) following the manufacturer’s protocol. Annexin V or PI-positive cells were considered apoptotic cells. For mitochondria analysis, cells were processed with a Mito-Traker Red kit (#C1035, Beyotime Biotechnology, China) per the manufacturer’s protocol. The samples were then analyzed by flow cytometer (Navios, Beckman Coulter, US).

### RNA-sequencing analysis

Total RNA was extracted from the cells using the TRIzol reagent (Invitrogen). The RNA was qualified and quantified using an Agilent 2100 bioanalyzer (Agilent Technologies, Palo Alto, CA, USA) and a NanoDrop instrument (ThermoFisher Scientific), respectively. Next-generation sequencing libraries were prepared using a NEBNext® Ultra™ RNA library prep kit for Illumina® according to the manufacturer’s protocol. RNA libraries with different indices were multiplexed and loaded on an Illumina HiSeq instrument (Illumina, San Diego, CA, USA). Sequencing was carried out using a 2′ 150-bp paired-end configuration. Gene set enrichment analysis (GSEA) was conducted using GSEA software, and a heatmap was prepared using online tools (https://software.broadinstitute.org/morpheus/). The raw data and normalized gene expression data were deposited in the Gene Expression Omnibus database under an accession number GSE182468.

### Reverse transcriptional quantitative PCR

The reverse transcriptional quantitative PCR (RT-qPCR) was performed as previously described [12]. Total RNA was extracted from MM cell lines using the TRIzol reagent (Invitrogen). Reverse transcription was performed using the *Evo M-MLV* RT kit (Accurate Biotechnology, China). RT-qPCR was performed using the SYBR Green qPCR master mix (Bimake, China). The primer sequences are listed in Supplementary Table 1. B2M is the reference gene of mitochondria DNA.

### Transmission electron microscopy

The cells were rinsed with PBS and fixed with glutaraldehyde in a cacodylate buffer. Osmication was done in 1% osmium tetroxide in a cacodylate buffer and dehydrated in an ascending series of ethanol solutions (25, 50, 75, 95%) for 10 min each, and in 100% ethanol two times for 10 min each. After dehydration, the cells were infiltrated with araldite:ethanol (1:1) for 2 h, araldite:ethanol (4:1) overnight, araldite for 45 min at 45 °C, araldite for 45 min at 55 °C, and araldite to a depth of 1.5–2 mm before polymerization at 60 °C overnight. Ultrathin sections (30 nm) of pale gold coloration cut from these blocks by an EM UC7 ultramicrotome (Leica, Inc., US) were collected onto 600-mesh copper grids, which were supported with Formvar films. The sections on the grids were examined in a HITACHI HT7700 transmission electron microscope.

### Animal study

For the murine 5T-MM mouse model, murine MM 5 TGM1 cells with consistent luciferase gene expression were intravenously injected into 6-weeks-old C57BL/KawRij mice (Harlan Co. Netherland) with 2 million cells per mouse. After tumor establishment, the mice were randomly divided into 4 groups. The tumor-bearing mice were treated with PBS (*n* = 10), melphalan only (*n* = 9; 60 μg per mouse every 2–3 days, intraperitoneal injection, for a total of 4 treatments), EGFR inhibitor gefitinib only (*n* = 10; 500 μg per mouse every 2–3 days, intraperitoneal injection, for a total of 4 treatments), or a combination of melphalan and gefitinib (*n* = 9). Three mice from each group were sacrificed 5 days after treatment and subjected to BM SP cell examination. The remaining mice were used to monitor treatment outcomes. Alternatively, after the 5T-MM mouse model was established, the tumor-bearing mice were divided into 3 groups and treated with PBS (*n* = 4), bortezornib (*n* = 6, 15 μg per mouse every 2–3 days, intraperitoneal injection, for a total of 4 treatments), or a combination of bortezornib and gefitinib (*n* = 6). The mice tumor burdens were examined by an in vivo luciferase assay using IVIS machinery (IVIS Spectrum In Vivo Imaging System, Perkin Elmer, US). Mouse peripheral blood was collected from the angular vein every 5 days. 5TGM1 monoclonal IgG2b protein levels in the peripheral blood were examined by ELISA kit (#88-50430-88, ThermoFisher Scientific, US). All mouse studies complied with protocols approved by the IACUC committee of West China Hospital, Sichuan University.

### Statistical analysis

Unless otherwise indicated, all data are expressed as the mean ± standard deviation. Statistical analysis was conducted using the statistical software GraphPad Prism 8. Survival was analyzed by the Kaplan–Meier log-rank test. A *p* < 0.05 was considered statistically significant.

## Results

### ALCAM regulates myeloma side population in bone marrow microenvironment

**Fig. 1:**
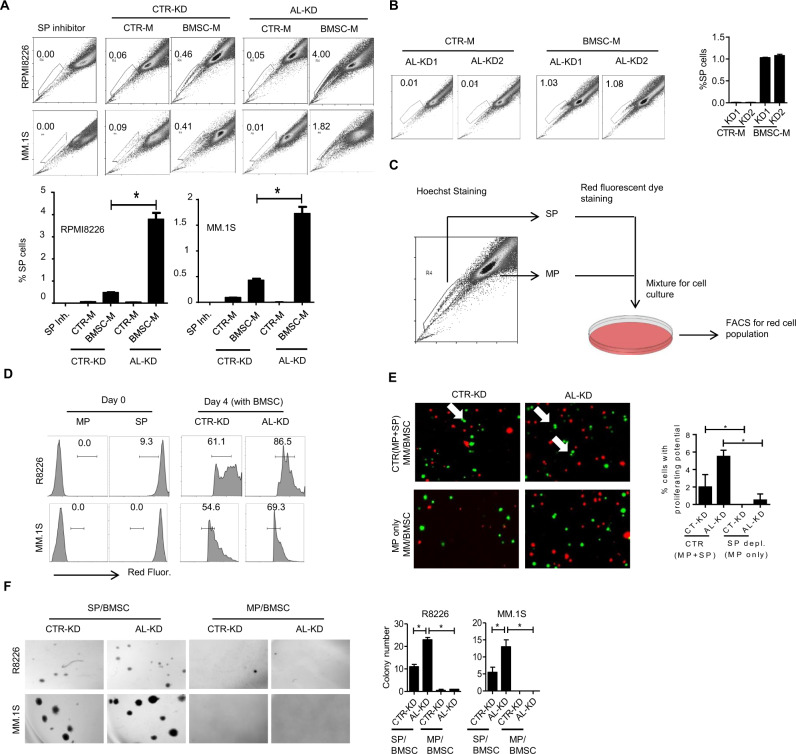
Bone marrow stroma-derived soluble factors promote ALCAM-knockdown myeloma cell side population. **A** Hoechst staining of CTR-KD or AL-KD MM cells (RPMI8226 or MM.1S) cultured in regular medium (CTR-M) or BMSC-conditioned medium (BMSC-M) for 48 h. The numbers indicate the percentage of SP cells. **B** Hoechst staining of AL-KD1 and AL-KD2 RPMI8226 cells cultured in regular medium (CTR-M) or BMSC-conditioned medium (BMSC-M) for 48 h. **C** Schematic graph: After cell sorting, SP cells were labeled with red fluorescent dye, and MP cells were labeled with CFSE. The labeled SP and MP cells were re-mixed with the ratio (SP:MP = 2:98). **D** SP cell proliferation was examined by flow cytometry for red fluorescent intensity dilution. **E** Fluorescence confocal microscopy of MM cells (green) and BMSCs (red) under 3D printing. **F** Hoechst staining of RPMI8226 cells, followed by cell sorting to isolate SP- and MP-only MM cells. The cells were used for the set of colony formation assay (left panel). Statistical results from the colony formation assays (right panel). Data are the mean of three independent experiments in three replicates. *p < 0.05.

Using shRNA lentivirus, we generated consistent ALCAM-knockdown MM cells (AL-KD), as well as control knockdown (CTR-KD). The knockdown efficacy was determined in our previous publication [12]. Hoechst staining showed that AL-KD MM cells (RPMI8226 and MM.1S) cultured in the BMSC-conditioned medium (BMSC-M) had a higher ratio of SP cells than CTR-KD (Fig. 1A). AL-KD cells generated with different ALCAM shRNA sequences showed a similar result (Fig. 1B). Next, we sorted SP MM cells after Hoechst staining and labeled the cells with CellTrace Far Red fluorescence dye (Fig. 1C). Since SP MM cells alone did not survive in vitro after sorting (data not shown), we mixed labeled SP cells with MP cells and cocultured the cell mix with BMSC for 4 days. AL-KD SP cells exhibited more “diluted” fluorescence intensity than CTR-KD SP cells, a result indicating that AL-KD SP had more active cell proliferation than CTR-KD SP under coculture (Fig. 1D). We also examined the clonogenic activities of MP and SP MM cells. We used a 3D printing technology to infuse MM cells (CFSE-labeled, green) and BMSCs (CellTrace Far Red-labeled, red) into a matrix. After coculture, SP-depleted MM cells had no MM proliferation identified (Fig. 1E). Finally, in a soft agar colony formation assay, only SP MM cells cocultured with BMSC resulted in colony formation. AL-KD SP cocultured with BMSC had more colony formation than CTR-KD SP cocultured with BMSC (Fig. 1F). Such findings agreed with a previous publication that SP MM cells exhibited tumor-initiating activity [14]. Overall, our results show that ALCAM-knockdown promotes SP MM cells in the presence of BM-derived factors. SP MM cells may contribute to tumor initiation.

### ALCAM-EGF/EGFR axis regulates myeloma side population

**Fig. 2:**
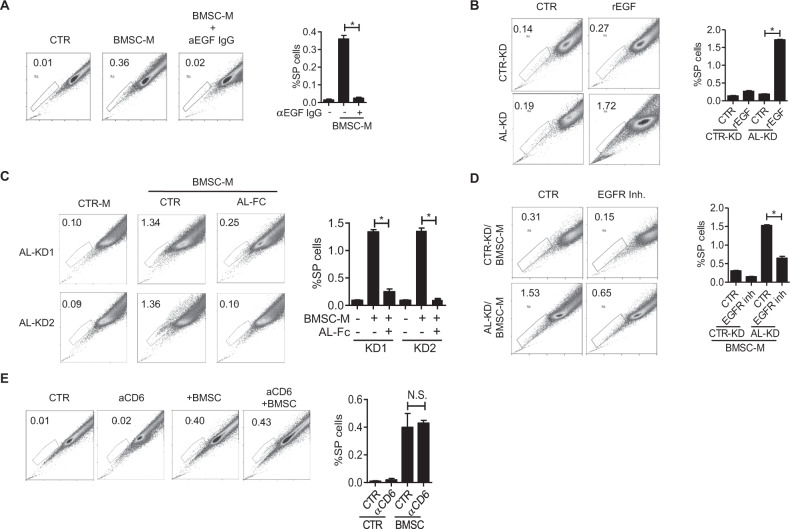
ALCAM-EGF/EGFR axis regulates myeloma side population. **A** Hoechst staining of RPMI8226 cells in the presence of an EGF neutralizing antibody (10 μg/ml) for 24 h and the result quantification (right panel). **B** Hoechst staining of CTR-KD or AL-KD RPMI8226 cells in the presence of recombinant EGF protein (10 ng/ml) for 24 h and the result quantification (right panel). **C** Hoechst staining of AL-KD1 and AL-KD2 RPMI8226 cells, cocultured with BMSC in the presence of ALCAM-Fc chimera fusion protein (AL-Fc, 0.5 μg/ml) for 24 h and the result quantification (right panel). **D** Hoechst staining of CTR-KD or AL-KD RPMI8226 cells, cocultured with BMSC in the presence of EGFR inhibitor (gefitinib, 200 nΜ) for 24 h and the result quantification (right panel). **E** Hoechst staining of RPMI8226 cells in the presence of CD6 antibody (10 μg/ml) and the result quantification (right panel). Data are the mean of three independent experiments in three replicates. *p < 0.05.

Previously, we showed that ALCAM interacts with EGFR and inhibits EGF priming-initiated EGFR downstream cell signaling and MM clonogenicity. Thus, we hypothesized that the ALCAM-EGFR/EGF axis regulated the abundance of SP MM cells. To test this hypothesis, we first examined the role of EGF in SP regulation. EGF blocking antibody inhibited SP population in MM cells cultured in BMSC-M (Fig. 2A), while the addition of EGF recombinant protein in the cell culture medium stimulated SP MM, particularly in AL-KD MM (Fig. 2B). Next, the addition of the ALCAM-Fc chimera fusion protein, which mimics the ALCAM function, inhibited BMSC-M stimulated SP population in AL-KD MM cells (Fig. 2C). EGFR inhibitor gefitinib inhibited the BMSC-M-stimulated SP population (Fig. 2D). Finally, we examined how the CD6 blockade affects SP MM. Previous research showed that ALCAM interacts with CD6 and mediates intercellular adhesion and migration [15]. However, the CD6 block antibody did not affect the BMSC coculture-stimulated SP population (Fig. 2E). Overall, our data confirm our hypothesis that the ALCAM-EGFR/EGF axis regulates the abundance of SP MM cells. Such regulation is independent of the ALCAM-CD6 interaction.

### ALCAM regulate myeloma side population through the hedgehog pathway

**Fig. 3:**
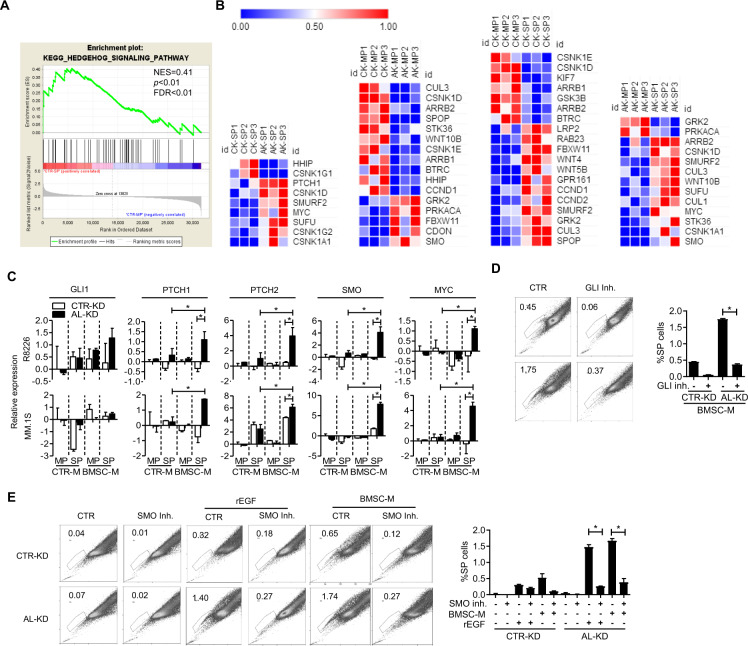
ALCAM suppresses the hedgehog pathway activation. **A** GSEA analysis showing the hedgehog pathway gene enrichment in SP and MP cells; **B** Heatmap showing differentially regulated hedgehog pathway gene expression. From left to right are comparisons of CK-KD SP vs. AL-KD SP, CK-KD MP vs. AL-KD MP, CK-KD MP vs. CK-KD SP, and AL-KD MP vs. AL-KD SP. *p* < 0.05 for all listed genes. **C** Reverse transcriptional quantitative PCR for hedgehog pathway gene expression in CTR-KD versus AL-KD MM cells. **D** Hoechst staining of CTR-KD or AL-KD RPMI8226 cells, cocultured with BMSC in the presence of GLI inhibitor (GNAT61, 10 μΜ) and the result quantification (below panel). **E** Hoechst staining of CTR-KD or AL-KD RPMI8226 cells, cocultured with BMSC or recombinant EGF protein (10 ng/ml) in the presence of SMO inhibitor (cyclopamine, 50 nM) for 24 h and the result quantification (right panel). Data are the mean of three independent experiments in three replicates. *p < 0.05.

Previous studies showed that SP MM cells may be regulated by hematopoietic stem cell regulatory cell signaling pathways such as the Wnt/β-catenin pathway [8], the hedgehog pathway [16], and the Notch pathway [17]. To identify ALCAM downstream cell signaling in SP MM regulation, we sorted SP and MP cells from CTR-KD and AL-KD MM in culture, and examined gene expression profiles. In general, MP and SP MM cells exhibited differentially regulated gene expressions (Supplementary Fig. 1A). Pathway enrichment analysis showed that the hedgehog pathway was altered in AL-KD MM cells, compared with CTR-KD (Fig. 3A). Differentially regulated hedgehog-pathway genes were identified (Fig. 3B). We verified some hedgehog pathway gene expression in MP versus SP cells by quantitative RT-PCR. As shown in Fig. 3C, SP cells isolated from the AL-KD MM cell culture had the highest hedgehog pathway gene expression. The hedgehog pathway inhibitor inhibited the BMSC-M- or EGF recombinant protein-stimulated SP population (Fig. 3D, E). We also analyzed the Wnt/β-catenin pathway and Notch pathway genes in MP versus SP MM cells, two pathways that have been shown to regulate MM tumor-initiating cells (Supplementary Fig. 1B, C). To summarize, our data suggest that the ALCAM-EGR/EGF axis regulates SP MM cells through the hedgehog pathway.

### The myeloma side population is chemoresistant

**Fig. 4:**
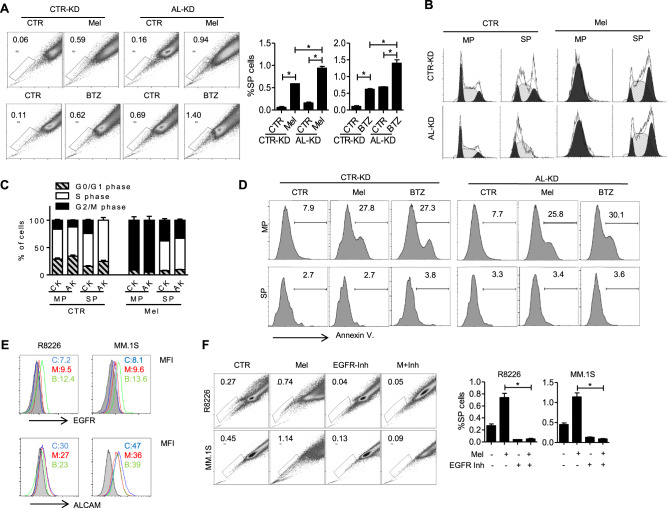
ALCAM regulates myeloma chemoresistant side population in vitro. **A** MM cells RPMI8226, either CTR-KD or AL-KD, were treated with melphalan (Mel, 15 μM) or bortezomib (BTZ, 5 nM) for 24 h. The SP cell ratio was examined by Hoechst staining. **B** The RPMI8226 cells were treated by melphalan as described above. The cell cycle was analyzed after Hoechst staining. **C** Cell-cycle quantification. **D** After Hoechst staining, the apoptotic cells were analyzed by annexin V staining. **E** ALCAM and EGFR expression on MM cells after Mel or BTZ treatment were detected by flow cytometry. MFI mean fluorescence index. **F** Examination of SP cells after EGFR inhibitor (gefitinib, 200 nM) and melphalan treatment. Data are the mean of three independent experiments in three replicates. *p < 0.05.

Previous research showed that SP MM cells are less sensitive than MP MM cells to chemotherapeutic agents. In our study, we found that anti-MM agents melphalan (Mel) or bortezomib (BTZ) treatment both induced an increased ratio of SP cells, particularly in AL-KD RPMI8226 MM cells (Fig. 4A). Cell-cycle analyses showed that, under the Mel treatment, SP cells still had an active cell cycle, while MP cells were arrested at the G2/M phase (Fig. 4B, C). The apoptotic assay showed that BTZ or Mel exposure could only induce apoptosis in MP cells, but not in SP cells (Fig. 4D). In our previous publication, as well as in data presented earlier in this study, we demonstrated that the ALCAM-EGFR/EGF axis regulated MM SP cells and clonogenicity. EGFR, primed with EGF, promoted MM SP cells, while ALCAM inhibited such promotion. The balance of EGFR and ALCAM expressions determined the SP ratio. Therefore, we examined ALCAM and EGFR expressions on MM cells after drug treatment. Mel or BTZ treatment-induced EGFR expression and repressed ALCAM expression (Fig. 4E). Thus, the drug-induced SP upregulation might occur due to the shift in the ALCAM and EGFR expression balance. Finally, we examined how the EGFR inhibitor gefitinib affects SP cells. We found that gefitinib inhibited melphalan-induced SP cells (Fig. 4F). We also repeated the above experiments in MM.1S cells and found similar results (Supple. Figure 2). Overall, our data show that anti-MM drug treatment induces the upregulation of chemoresistant SP MM cells. EGFR inhibition blocks chemoresistant SP MM cell generation.

### Myeloma side population cells have increased the number of mitochondria

**Fig. 5:**
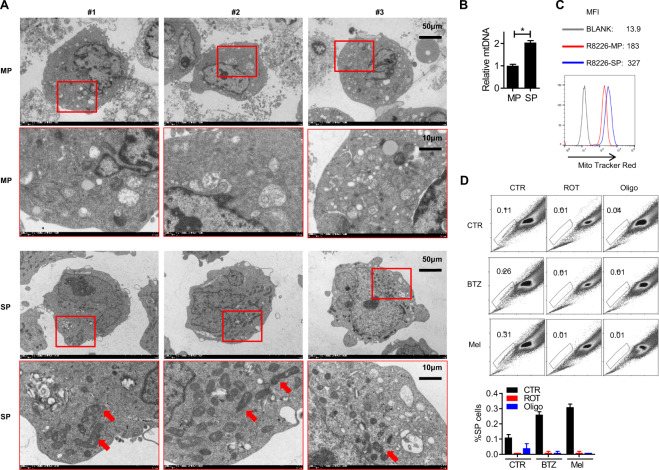
Myeloma side population cells have increased the number of mitochondria. **A** Transmission electron microscopy images of RPMI8226 SP and MP cells. **B** Mitochondria DNA (mtDNA) of RPMI8226 SP and MP cells were examined by qPCR. **C** Mito-tracker staining of RPMI8226 SP and MP cells. MFI mean fluorescence index. **D** RPMI8226 MM cells were treated with oligomycin (Oligo, 3 μM) or rotenone (ROT, 1 μM) for 4 h. The SP cell ratio was examined by Hoechst staining. Data are the mean of three independent experiments in three replicates. *p < 0.05.

Next, we characterized the morphological features of SP MM RPMI8226 cells. Transmission electron microscopy (TEM) showed that SP cells had significantly more mitochondria than MP cells (Fig. 5A). In addition, mitochondria DNA (mtDNA) examination (Fig. 5B) and mito-tracker staining (Fig. 5C) both suggested that SP cells had more mitochondria than MP cells. Inhibition of the mitochondria functions strongly inhibited drug-induced SP cell upregulation (Fig. 5D). To summarize, our data show that mitochondria play a critical, but still mechanistically undetermined, role in SP MM cells.

### EGFR-targeting therapy overcomes myeloma side population-mediated drug-resistance in vivo

**Fig. 6:**
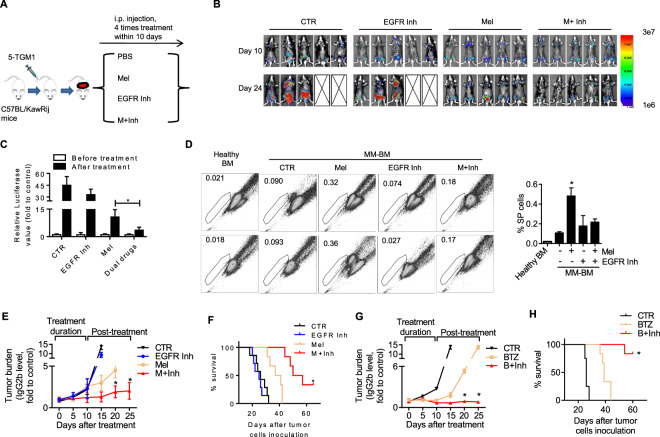
EGFR-targeting therapy attenuates side population conferred myeloma chemoresistance in vivo. **A** Scheme graph showing the animal study to evaluate the efficacy of the combination therapy (Mel and EGFR inhibitor) in vivo. The mice were treated by intraperitoneal injection of melphalan (60 μg/mouse per time, 4 times within 10 days) or gefitinib (500 μg/mouse per time, 4 times within 10 days), or a combination of both. Each group contained 8 mice. **B** Tumor-bearing mice were subjected to in vivo bioluminescent imaging (IVIS) before and after treatment. Five out of seven representative results are shown. **C** The relative luciferase activity of IVIS was calculated. **D** The tumor-bearing mice were treated twice as described above. Then, mice BM cells were analyzed by Hoechst staining for MM SP in vivo. Two out of three representative results are shown in the left panel, and result quantification is shown in the right panel. **E** Tumor burden was evaluated by circulating IgG2b. **F** Treatment efficacy was evaluated by mouse survival. **G** MM tumor-bearing mice were treated by intraperitoneal injection of PBS, bortezomib (15 μg/mouse per time, 4 times within 10 days), or a combination of bortezomib and gefitinib (500 μg/mouse per time, 4 times within 10 days). The PBS group contained 4 mice; the other treated group each contained 6 mice. Tumor burden was evaluated by circulating IgG2b. **H** Treatment efficacy was evaluated by mouse survival (*p < 0.05).

According to the above data, we proposed that anti-MM drug treatment would induce increased chemoresistant SP MM cells. ALCAM-low MM had a higher capacity than ALCAM-high MM cells in generating SP cells. Therefore, ALCAM-low MM might be more resistant to anti-MM agents than ALCAM-high MM. Next, we examined SP-mediated MM chemoresistance in vivo in a 5T-MM model (Fig. 6A). Tumor imaging showed that EGFR inhibition alone, which could block SP cell generation, did not affect tumor growth in vivo. Melphalan and EGFR inhibitor gefitinib combination therapy resulted in better tumor shrinkage than melphalan alone (Fig. 6B, C). Analyses of ex vivo tumor cells showed that gefitinib repressed SP MM cells (Fig. 6D). Circulating M protein (Fig. 6E) and mice survival (Fig. 6F) both showed that combination therapy had a better treatment outcome compared to single-drug treatment. In particular, the combination-treated mice had a prolonged stage of stable disease after treatment. The same was true in BTZ-based treatment (Fig. 6G, H). To summarize, we showed that SP MM cells mediate drug-resistance in vivo. EGFR inhibition can therefore overcome SP-mediated MM chemoresistance.

### ALCAM expression in primary myeloma cells

**Fig. 7:**
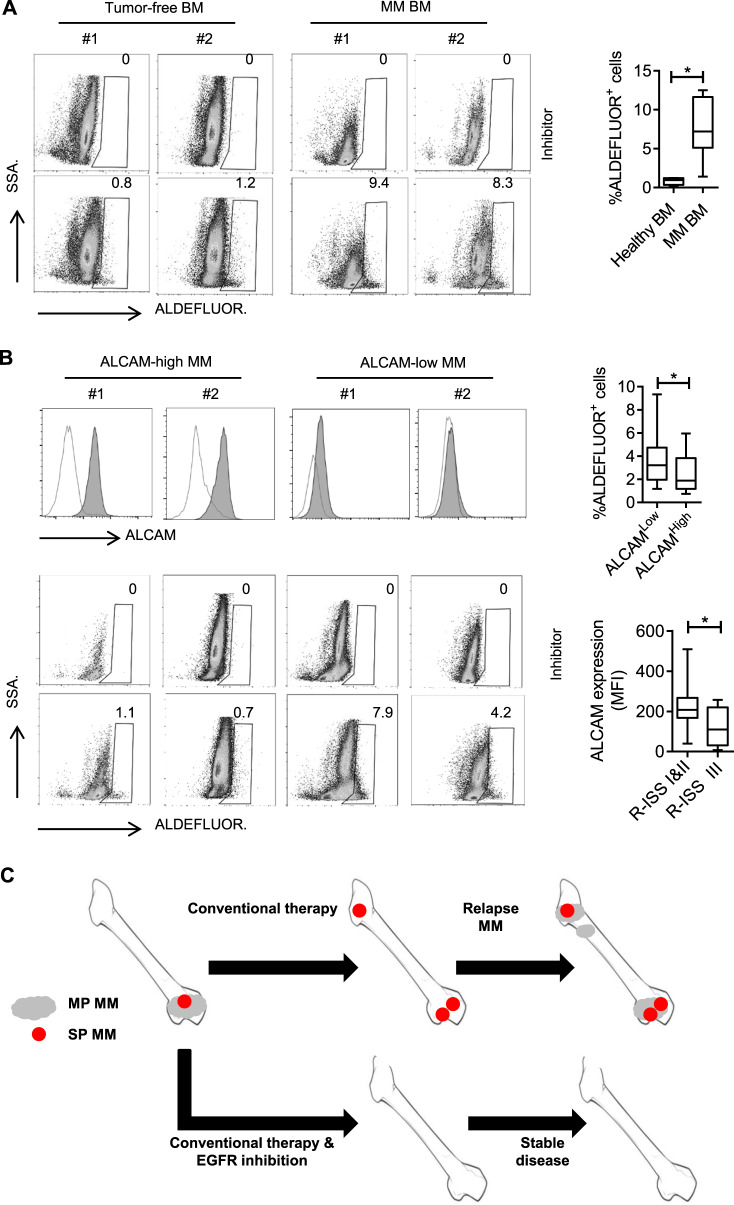
ALCAM expression and stemness myeloma cells. **A** ALDEFLUOR analysis of BM cells from non-tumorous donors (*n* = 5) and MM patients (*n* = 7). Two representative results are shown. **B** BM cells from 42 newly diagnosed MM patients were analyzed by flow cytometry for ALCAM expression in CD138^+^ MM cells and ALDEFLUOR staining. Results show 2 representatives of ALCAM-high MM versus ALCAM-low MM, and corresponding ALDEFLUOR staining results. **C** Graphic model of EGFR-targeting therapy in MM treatment (*p < 0.05).

Finally, we examined the stemness of MM cells in primary samples. Since BM aspiration from each patient could not provide sufficient cells for Hoechst staining, we used an ALDEFLUOR assay to determine stemness cells in BM. The ALDEFLUOR assay identified cancer stem cells in vitro and in vivo based on the cell aldehyde dehydrogenase activity [18]. As shown in representative data in Fig. 7A (left) and the summary of results (Fig. 7B, right), MM BM had a higher ratio of ALDFLUOR^+^ cells than normal BM. Next, we performed an ALDEFLUOR assay in a group of primary MM cells. The patient characteristics are summarized in Supplementary Table 2. ALCAM expression in primary MM cells was determined by RT-qPCR. The patients were divided into two groups according to ALCAM expression. The patients with ALCAM^Low^ MM had a higher ratio of ALDFLUOR^+^ cells than patients with ALCAM^High^ MM (Fig. 7B); R-ISS III MM patients had more ALDFLUOR^+^ cells than R-ISS I&II patients (Fig. 7B). To summarize, our data show the existence of ALDFLUOR^+^ in MM BM. The ratio of ALDEFLUOR^+^ cells in MM BM negatively correlates with MM ALCAM expression.

## Discussion

Previously, we identified functional crosstalk between ALCAM and EGFR [12]. MM cells expressed both ALCAM and EGFR. ALCAM interacted with EGFR and blocked BM microenvironment-derived EGF binding to its receptor, thus inhibiting the EGFR downstream cell signaling that regulated MM clonogenicity. In this study, we further investigated the ALCAM-EGF/EGFR axis in MM drug-resistance regulation. We found that the level of ALCAM expression on MM cells correlated with the ratio of drug-resistant SP MM cells within the BM microenvironment. Anti-MM agent treatment stimulated SP MM cell generation in vitro and in vivo. ALCAM^High^ MM cells had lower SP-genesis capacity than ALCAM^Low^ MM cells. The low SP-genesis capacity of ALCAM^High^ MM was consistent with our previous findings that ALCAM^High^ MM exhibited low clonogenesis and prolonged patient survival [12].

The MM drug-resistance mechanism is complex and still under active investigation. Some researchers have introduced the concept of MM cancer stem cells (MM-CSCs) to demonstrate drug-resistance. In general, CSCs refer to a small population of malignant cells with distinguishable activities of clonogenicapacity, self-renewal, and differentiation into regular cancer cells [19]. Previous studies indicated that MM-CSCs were resistant to chemotherapy and might be a promising target to control drug-resistance [8, 20]. In many ways, MM-CSCs are still conceptual, so there has been controversy in defining and characterizing them [20]. Alternative terminologies might be used for describing those cells, such as MM stemness side population [7], MM clonogenic cells [21], or MM cancer-initiating cells [22]. Although MM-CSCs have not yet been properly defined, there are still several ways to detect them, such as via Hoechst staining [20] and an ALDEFLUOR assay [18], both of which were used in our study.

It is generally believed that CSCs may have important therapeutic implications [23]. Our results suggest that a combination therapy with an EGFR inhibitor and anti-MM agents might be promising for MM treatment. EGFR inhibitor alone has no anti-MM activity in vitro and in vivo. A previous cell line study suggested that EGFR inhibitor gefitinib had no anti-MM activity against MM cells with KRAS, NRAS, or BRAF mutations [24]. An earlier phase II study of VEGFR/EGFR inhibitor Zactima in MM also showed that the inhibitor was well tolerated in patients but could not reduce the patients’ M protein [25]. According to our findings, EGFR inhibition repressed drug-resistant SP MM cells, which only accounted for a small number of total neoplasmic cells. Therefore, EGFR inhibitor should be used in combination with other anti-MM agents. Our findings also suggest that, during the combination therapy, anti-MM agents were used to eradicate most MM cells, while EGFR inhibition targeted a small number of drug-resistant MM cells. Combination therapy might increase the duration of complete remission after treatment and decrease the frequency of treatment. To the best of our knowledge, there are no ongoing clinical trials testing EGFR inhibition and anti-MM combination therapy. This lack of research is probably because EGFR expression is low and EGFR gene mutation is rare in MM. Our findings provide evidence to support such clinical studies. Further, we also believe that MRD status after treatment and CR duration time are the two critical factors needed to evaluate the combination therapy.

## Supplementary information

Supplement information

Supplement information 2

checklist

## Data Availability

The data sets generated and/or analyzed during the current study are available from the corresponding author on reasonable request.
