# Supplementary material for: ALCAM regulates multiple myeloma chemoresistant side population
Source: Cell Death Dis. 2022 Feb 10;13(2):136. doi: 10.1038/s41419-022-04556-8 (PMC8831486; doi:10.1038/s41419-022-04556-8)
Supplement: Supplementary file 1 — Supplement information [file 41419_2022_4556_MOESM1_ESM.pdf]

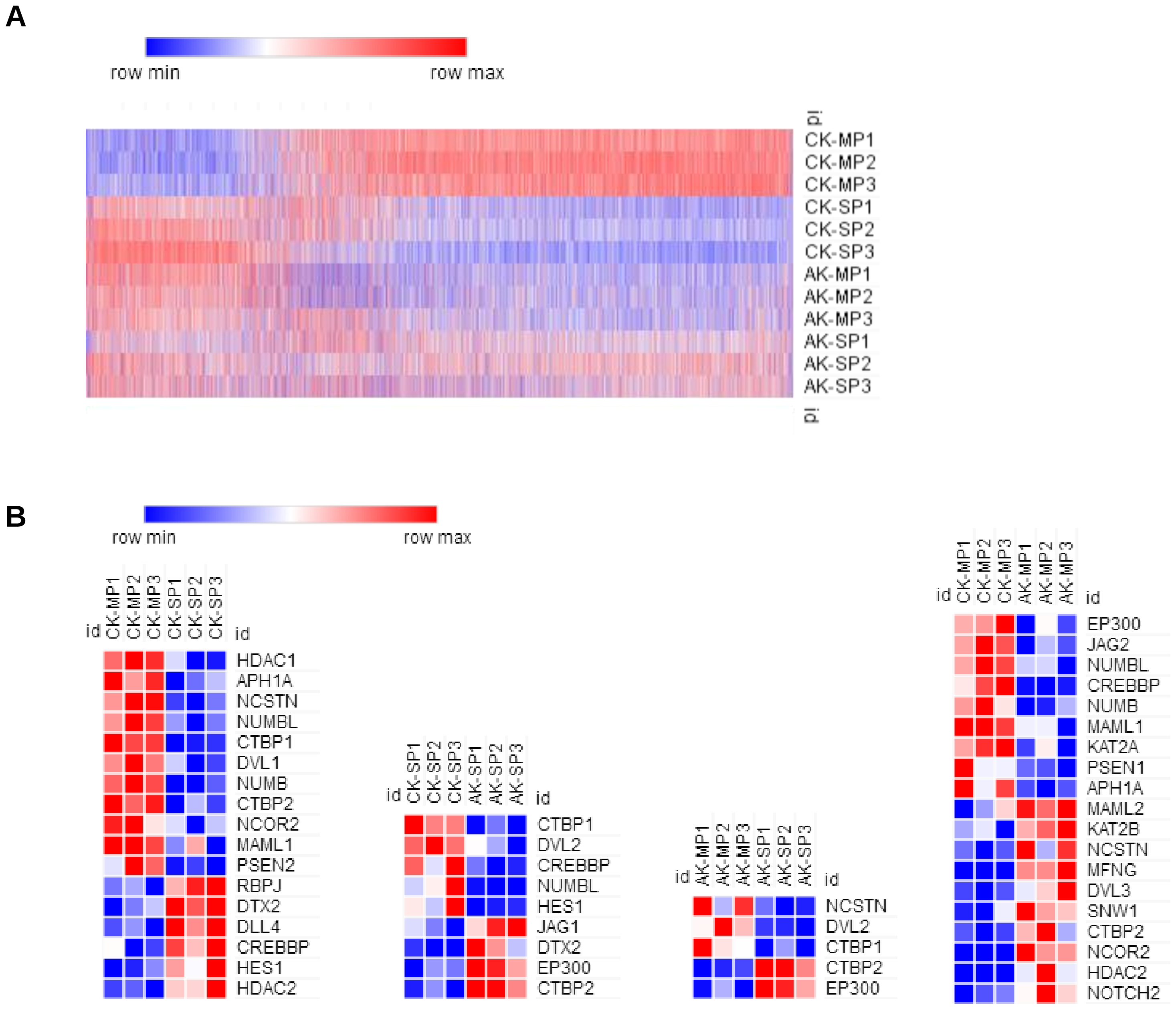

Supplementary Figure 1. Main population and side population myeloma cells have differentially regulated gene expression

C

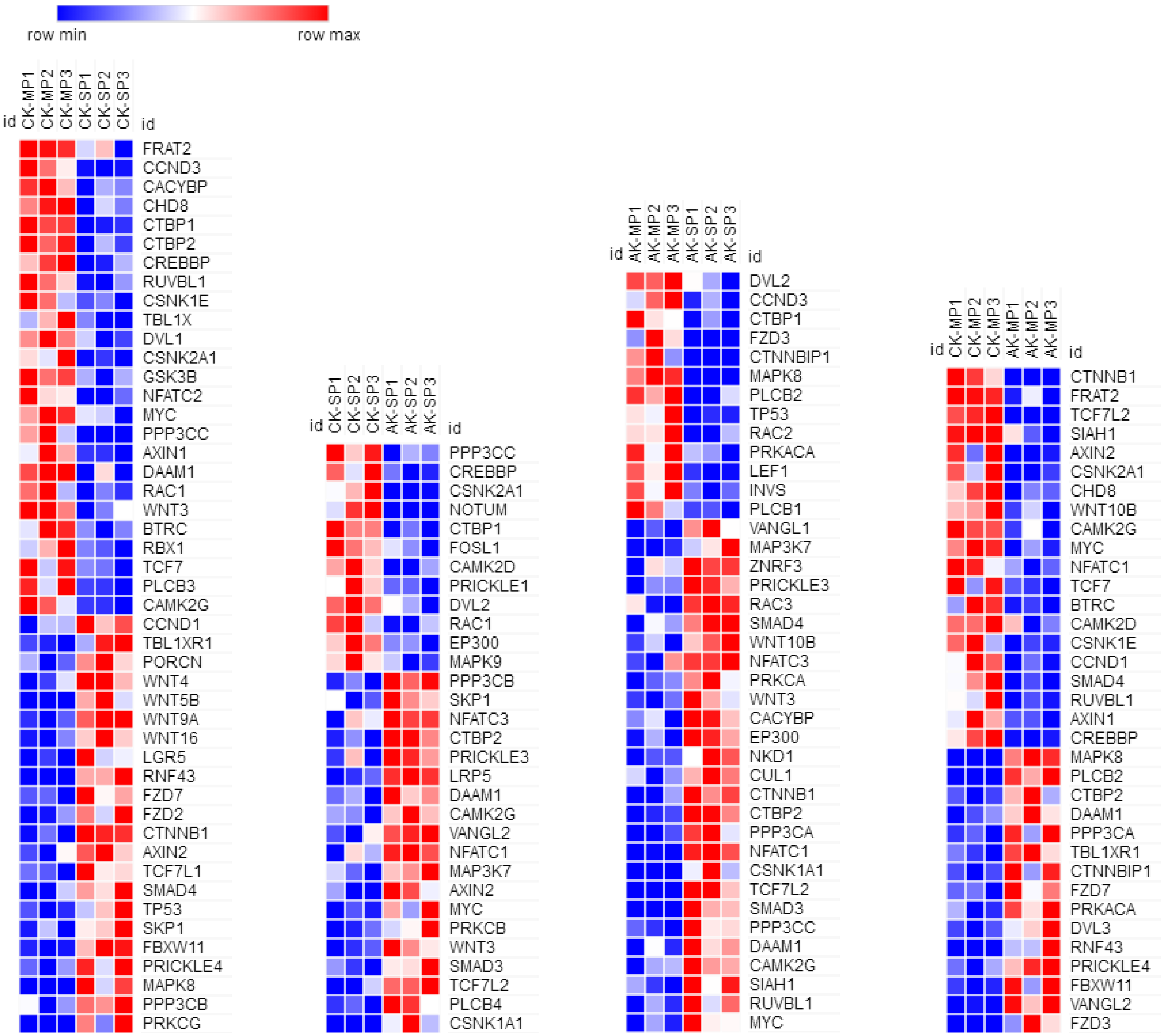

Supplementary Figure 1. Main population and side population myeloma cells have differentially regulated gene expression

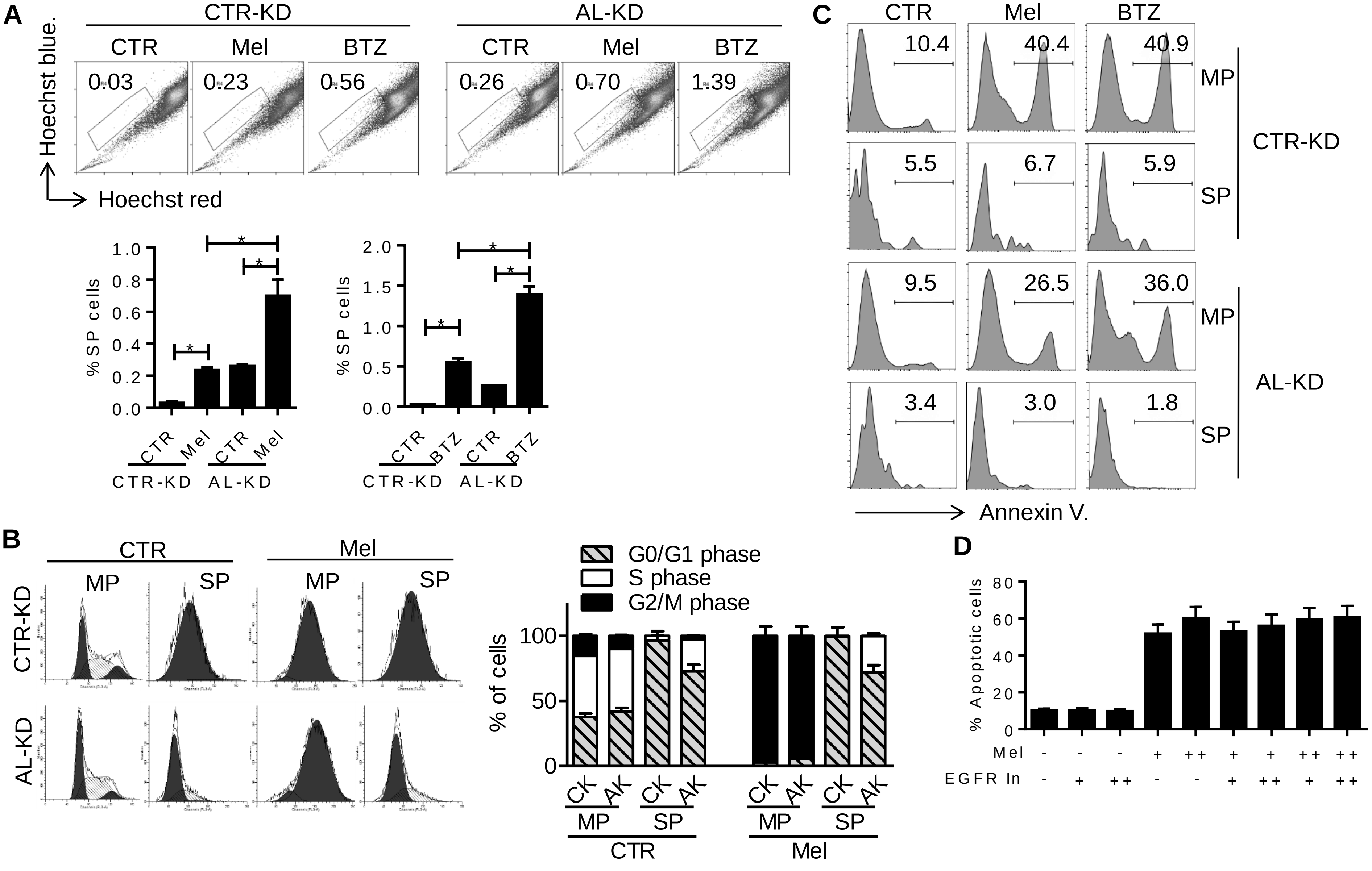

Supplementary Figure 2. Side population-mediated drug-resistance in MM.1S

GENE		PRIMERS
ALCAM	Forward	ACTTGACGTACCTCAGAATCTCA
	Reverse	CATCGTCGTACTGCACACTTT
GLI1	Forward	AGCCTTCAGCAATGCCAGTGAC
	Reverse	GTCAGGACCATGCACTGTCTTG
PTCH1	Forward	GCTGCACTACTTCAGAGACTGG
	Reverse	CACCAGGAGTTTGTAGGCAAGG
PTCH2	Forward	GCACTATTACCGCAACTGGCTAC
	Reverse	TCTCCAGTCTGGATGAGCAGCT
MYC	Forward	CCTGGTGCTCCATGAGGAGAC
	Reverse	CAGACTCTGACCTTTTGCCAGG
SMO	Forward	AATGCGTGCTTCTTTGTGGG
	Reverse	TCTCATTGGAGGTGGGCTCC
mtDNA	Forward	GTCAACCTCGCTTCCCCACCCT
	Reverse	TCCTGCGAATAGGCTTCCGGCT
B2M	Forward	TGCTGTCTCCATGTTTGATGTATCT
	Reverse	TCTCTGCTCCCCACCTCTAAGT
GAPDH	Forward	GTCTCCTCTGACTTCAACAGCG
	Reverse	ACCACCCTGTTGCTGTAGCCAA

Supplementary Table 1. Quantitative PCR primers

Variable	ALCAM ^{high}	ALCAM ^{low}	p value
	n=21	n=21	
Median age,y (range)	61（50-78）	62（42-76）	0.544
Female,no.(%)	8(38.1)	13(61.9)	0.123
Secondary or treatment related MM,no. (%)	2（9.5）	0	0.469
RISS stage,no.(%)			0.052
Ⅰ	3(14.3)	2（9.5）	
Ⅱ	11(52.4）	8（38.1）	
Ⅲ	4（19）	10（47.6）	
NA	3	1	
Median hemoglobin,g/L (range)	121（60-156）	108（62-137）	0.09
Median platelet,10^9 (range)	194（55-332）	158（76-352）	0.468
Median WBC, 10^9(range)	6.22（2.45-24.51）	5.77（2.54-12.2）	0.273
Median albumin,g/L (range)	40.9（26.1-52.5）	37.2（0-52.8）	0.143
Median globulin,g/L (range)	33.5（16.6-74.5）	47.1（13.6-113.9）	0.223
Median LDH,IU/L (range)	181.5（118-255）	189（97-508）	0.34
Median creatinine,umol/L (range)	67（54-412）	78（34-822）	0.397
Median eGFR,ml/min/1.73m^2 (range)	91（8.47-108.38）	79.14（7.43-126.05）	0.673
Median M-protein,% (range)	7.9（0-50.2）	26.6（0-61.2）	0.25
Median serum beta-2 microglobulin,mg/L (range)	2.7（1.47-21.8）	4.76（1.57-26.5）	0.237
Chromosomal abnormalities,no.(%)			0.513
Standard-risk	4（19）	3(14.3)	
High-risk	1（4.7)	2(9.5)	
NA	16	16	

WBC:white blood cell
LDH:lactate dehydrogenase
eGFR:estimated glomerular filtration rate
Standard-risk:No high-risk chromosomal abnormalities
High-risk:del(17/17p),t(4;14),t(14;16)

Supplementary Table 2. Characteristics of newly diagnosed myeloma patients
