# Supplementary material for: ALCAM regulates multiple myeloma chemoresistant side population
Source: Cell Death Dis. 2022 Feb 10;13(2):136. doi: 10.1038/s41419-022-04556-8 (PMC8831486; doi:10.1038/s41419-022-04556-8)
Supplement: Supplementary file 2 — Supplement information 2 [file 41419_2022_4556_MOESM2_ESM.docx]

**Supplementary Figure Legends**

**Supplementary Figure 1. Main population and side population myeloma cells have differentially regulated gene expression**

**A** Heatmap showing the differentially regulated genes in MP and SP cells of CTR-KD and AL-KD R8226 cells; **B** Heatmap showing the differentially regulated Notch pathway genes in tested samples. From left to right were comparisons of CK-KD MP vs. CK-KD SP, CK-KD SP vs. AL-KD SP, AL-KD MP vs. AL-KD SP, and CK-KD MP vs. AL-KD MP. *p*<0.05 for all listed genes; **C** Heatmap showing the differentially regulated Wnt/b-catenin pathway genes. From left to right are comparisons of CK-KD MP vs. CK-KD SP, CK-KD SP vs. AL-KD SP, AL-KD MP vs. AL-KD SP, and CK-KD MP vs. AL-KD MP. p<0.05 for all listed genes.

**Supplementary Figure 2. Side population-mediated drug-resistance in MM.1S**

**A** MM cells MM.1S, either CTR-KD or AL-KD, were treated with melphalan (Mel, 15 μM) or bortezomib (BTZ, 5 nM) for 1 day. The SP cell ratio was examined by Hoechst staining. **B** The MM.1S cells were treated by melphalan as described above. The cell cycle was analyzed after Hoechst staining. Cell cycle quantification. **C** After Hoechst staining, the apoptotic cells were analyzed by annexin V staining. **D** The apoptotic cells of MM.1S cells were treated by melphalan (Mel, +: 10μM; ++:15 μM), EGFR inhibitor (EGFR In, gefitinib, +: 200 nM; ++: 400 nM) or their combination.

**Supplementary Table Legends**

**Supplementary Table 1. Quantitative PCR primers. Primers used in quantitative PCR.**

**Supplementary Table 2. Characteristics of newly diagnosed myeloma patients**
